# The use of Bayesian methods for the analysis of Studies Within A Trial: a proof-of-concept case study

**DOI:** 10.1186/s13063-026-09726-z

**Published:** 2026-04-24

**Authors:** Suzie Cro, Zhangyi He, Daphne Babalis, Laurent Billot

**Affiliations:** 1 Imperial Clinical Trials Unit, Imperial College London, 1st floor, Stadium House, White City, London, W12 7RH England, UK; 2 The George Institute for Global Health, Imperial College London, London, England, UK; 3 The George Institute for Global Health, University of New South Wales, Sydney, NSW Australia

**Keywords:** Study within a trial, SWAT, Bayesian, ACCEPT, Statistical analysis

## Abstract

**Background and Aims:**

A Study Within A Trial (SWAT) is a research study embedded within a larger trial which aims to investigate different strategies for a particular trial process, such as trial recruitment. It is imperative such studies, which are often underpowered, employ efficient and informative analytical methods. The use of Bayesian methods and interpretation of SWAT results in this context requires exploration. Bayesian methods provide direct probability statements about intervention effects and readily enable ACceptability Curve Estimation using Probability above Threshold (ACCEPT) analyses, which consider the probability of the tested intervention being effective for different threshold values. Additionally, they provide an opportunity to incorporate the results of previous similar studies within the analysis using informative priors. This proof-of-concept study re-analysed two previous SWATs using Bayesian methods and ACCEPT analyses.

**Methods:**

A SWAT conducted by Du et al. in 2009 and a subsequent SWAT by Mattock et al. in 2020 compared a video intervention against standard patient information on trial recruitment. For each SWAT, a primary Bayesian analysis was performed using a logistic model with non-informative priors. Sensitivity analysis explored informative priors informed by meta-analysis of previous similar studies; this included an analysis of the Mattock et al. SWAT incorporating the result of the earlier Du et al. SWAT. ACCEPT curves were constructed. Results were compared with frequentist analyses.

**Results:**

For the Du et al. SWAT, the primary Bayesian analysis gave an OR for recruitment for the video relative to standard information of 2.12, 95% CrI: 0.38–4.65 and a posterior probability of the video being effective (OR > 1) of 0.86. When taking into account results of previous SWATs by using an informative prior there remained a moderately high probability of video benefit (0.82). For the latter Mattock et al. SWAT, the primary Bayesian analysis gave an OR for recruitment for the video relative to standard information of 0.26, 95% CrI: 0.07–0.51 and the posterior probability of the video being effective was 0.0005, indicating very little chance of effectiveness; ACCEPT plots facilitated interpretation by showing the probability that the video was better than standard information for OR > 0.8 was very small (0.0032). When taking into account the results of previous SWATs using an informative prior, including Du et al., the probability of the video being effective was still very small (0.12).

**Conclusions:**

Bayesian methods and ACCEPT analyses offer solutions to challenges experienced in the analysis and interpretation of SWATs, which are often underpowered. Greater use of these analytical approaches within SWATs will lead to a more accessible, improved evidence base on how to effectively conduct trials.

**Supplementary Information:**

The online version contains supplementary material available at 10.1186/s13063-026-09726-z.

## Background and Aims

Randomised Controlled Trials (RCTs) provide vital evidence on the efficacy, effectiveness and safety of new and existing interventions. Despite often being costly and resource heavy [1], there is sparse evidence to support decisions on how to best conduct randomised trials [2]. For example, one common issue identified as an important conduct challenge to address is trial recruitment [3]. A Study Within A Trial (SWAT) is a research study embedded within a larger host trial which aims to investigate different strategies for a particular trial process, such as trial recruitment [2].

Previous SWATs that have addressed the issue of recruitment include a SWAT by Du et al. (2009) [4] which tested whether an educational video intervention improved recruitment over standard patient information for a breast cancer trial. Although trial enrolment was higher in the video intervention group, the difference was not statistically significant. Subsequently the Healthy Start, Happy Start (HSHS) SWAT by Mattock et al. (2020) [5] tested whether adding a participant information video clip to a standard Participant Information Sheet (PIS) improved recruitment into the HSHS trial. This found no benefit of a video intervention.

Relatively small numbers of SWATs have been conducted, but SWAT numbers are now increasing [6], so it is imperative such trials employ efficient and informative analytical methods to enable robust inference and conclusions. Like their main host RCTs, SWATs are typically analysed using a frequentist statistical framework (see summary Table 1). This usually entails conducting a hypothesis test to obtain a *p*-value which leads to a binary conclusion of an intervention effect or not. The p-value represents the probability of obtaining data at least as extreme as what was observed, assuming the null hypothesis of no intervention effect is true over repeated trials. A small *p*-value (typically < 0.05) provides evidence against the null hypothesis. For example, a *p*-value of *p* = 0.03 means that if there were truly no intervention effect then results at least as extreme as those observed would occur about 3% of the time in repeated trials. But this definition may not be easily intuitive to non-statisticians. This is quite different to the probability that the intervention does not work.

**Table 1:** Frequentist versus Bayesian inference for SWATs

	**Frequentist inference**	**Bayesian inference**
General approach to inference	Treats the intervention effect as fixed but unknown, and inference is based only on the observed SWAT data	Treats the intervention effect as a random variable, combines prior information about the intervention effect (in the form of a prior distribution) with the observed SWAT data resulting in a posterior distribution for the intervention effect for inference
Intervention effect estimate	Provides an estimate of the intervention effect, e.g. odds ratio	Provides an estimate of the intervention effect, e.g. odds ratio
Use of prior information	Prior information (or beliefs) are not explicitly incorporated — inferences are based only on the SWAT data at hand	Prior information (or beliefs) can be explicitly incorporated through the prior distribution. The prior is combined with the SWAT data at hand for inference. The prior can be vague/uninformative with the absence of any other prior information
Quantifying uncertainty	Uncertainty in the intervention effect is quantified by a confidence interval (CI), typically a 95% CI, which represents the range of values for the intervention effect that with a large number of repeated samples, 95% of such calculated confidence intervals would include the true value of the intervention effect	Uncertainty in the intervention effect is quantified by a credible interval (CrI), typically a 95% CrI, which represents the region that the intervention effect falls within with 95% probability according to the posterior distribution
Hypothesis testing and inference	Hypothesis testing is based on *p*-values. The *p*-value represents the probability of obtaining data at least as extreme as what was observed assuming the null hypothesis of no intervention effect is true. The *p*-value is used to determine whether there is evidence to reject the null hypothesis or not	Inference is based on posterior probabilities, which quantify the relative likelihood of particular hypotheses, for example the probability of an intervention being effective or the probability of an intervention being effective by a specified amount compared to a control

To avoid making an incorrect conclusion from a hypothesis test, a trial needs to be adequately sized. If the sample size is too small the trial will have low statistical power, meaning a low chance of detecting an intervention effect size of interest when it truly exists; there will be an increased chance of random variation leading to an incorrect false negative conclusion. However, since the sample size available is typically dictated by the host trial, SWATs are often (but not always) small or underpowered for the SWAT outcome of interest. Since SWATs are not always adequately powered, there is a risk that incorrect conclusions may be reached and an effective intervention missed by adopting frequentist inference and focusing on *p*-values.

An alternative analytical approach is to adopt Bayesian inference. Bayesian methods combine prior information about the intervention effect (in the form of a prior distribution) with the observed trial data resulting in what is referred to as a posterior distribution for the intervention effect for inference. This approach has several additional advantages. Firstly, Bayesian methods provide an alternative interpretation of trial data in the form of direct statements on the probability of the intervention being effective, which can be more intuitive to understand than *p*-values (see summary Table 1) [7–9]. For example, a Bayesian analysis may conclude that there is a 90% probability that an intervention is effective compared to a control (i.e. 90% probability OR > 1). Alongside *p*-values, frequentist analysis will typically report a 95% confidence interval to quantify the uncertainty for the intervention effect of interest, but a 95% confidence interval for an intervention effect does not mean there is a 95% chance that the true intervention effect is in the given interval. It means that over repeated hypothetical trials, 95% of such intervals would be expected to contain the true value. To quantify uncertainty in estimation in Bayesian analyses, typically a 95% credible interval (CrI) is reported which represents the region that the intervention effect falls within with 95% probability according to the posterior distribution. For these nuisances in interpretation, Bayesian methods can be more initiative for non-statisticians to fully understand.

By producing a posterior distribution for the intervention effect of interest, more nuanced questions can readily be asked. For example, one can consider the probability that the intervention is effective and the probability that the intervention effect is clinically meaningful by different specified amounts. Frequentist trials focus on hypothesis testing and *p*-values and generally result in a binary verdict. But Bayesian methods can readily provide richer insights rather than just significant or non-significant.

Bayesian methods also enable prior knowledge to be combined with current trial data. This means that where relevant, analysis can be supplemented by existing knowledge from earlier studies or expert belief. For this reason, Bayesian methods are also particularly suited to smaller sample size settings, due to the ability to incorporate external data, for example from previous similar studies [7, 10]. This can lead to more efficient analysis with less uncertainty around estimates. But of course, prior information incorporated into a Bayesian analysis must be carefully justified and sensitivity around the prior information is recommended.

Despite the aforementioned advantages, the application of Bayesian methods and how to interpret SWAT results in this context has not been widely explored. Additionally, ACceptability Curve Estimation using Probability above Threshold (ACCEPT) analyses, which consider the probability of the intervention effect being effective for various different thresholds, have been proposed as a way of maximising the utility of trial data and are readily conducted in a Bayesian framework [11]. ACCEPT has only been used sporadically in clinical trials [11] and has not yet been applied to SWATs.

The aim of this proof-of-concept study was to re-analyse the SWATs by Du et al. and Mattock et al. using Bayesian methods to explore the utility of Bayesian methods for the analysis of SWATs. We applied ACCEPT analyses to demonstrate the accessible interpretation Bayesian methods enable and explore how previous external data can be incorporated within the analysis to strengthen conclusions. The resulting Bayesian inference was compared to that of frequentist analyses.

## Methods

### Description of Du et al. 2009 SWAT

Du et al. previously conducted a SWAT to examine the effect of an 18-min educational video compared to standard patient information on enrolment rates for a breast cancer trial [4]. A total of 196 eligible patients scheduled for treatment evaluation at a breast cancer clinic in the USA were contacted by telephone and consented to participate in the SWAT and were randomised to view either the educational video about cancer clinical trials prior to their first clinic appointment (*n* = 98) or to standard patient information (*n* = 98). The primary outcome of the SWAT was participants’ recruitment status into the host breast cancer treatment clinical trial. Full details of the SWAT and trial methods have been published previously [4].

The original trial used frequentist statistical methods for analysis including the Chi-square and Fisher’s exact test. Results were presented using odds ratios (ORs), *p*-values, and 95% confidence intervals (CIs) to compare the recruitment intervention strategy. A 95% CI represents the range of values for the intervention effect that with a large number of repeated samples, 95% of such calculated confidence intervals would be expected to include the true value of the intervention effect. A *p*-value provides evidence of data incompatibility with the null hypothesis of no intervention effect, rather than evidence of the alternative hypothesis of an intervention effect. We replicated the frequentist analysis using a logistic regression model including intervention as a covariate.

### Description of Mattock et al. HSHS SWAT

The HSHS study was a UK-based RCT which assessed the effectiveness and cost-effectiveness of a Video-feedback Intervention to promote Positive Parenting and Sensitive Discipline (VIPP-SD) in preventing enduring behavioural problems in young children aged 12–36 months [12]. The embedded HSHS SWAT conducted by Mattock et al. was conducted after the Du et al. SWAT and tested whether adding a participant information video clip to a standard PIS improved recruitment into the HSHS trial [5]. In total 107 out of 317, potential HSHS participants were included in the SWAT; 51 randomised to the PIS group and 56 to the information video clip, but six participants were excluded from analysis due to being inadvertently allocated to the wrong group, resulting in 101 in the analysis population (*n* = 51 PIS and *n* = 50 video clip).

The primary outcome of the Mattock et al. HSHS SWAT was the proportion of participants who consented to recruitment in the host trial based upon their randomised SWAT allocation. The original analysis was conducted using frequentist methods. Specifically, a logistic regression model was used to examine the effect of SWAT allocation (video vs PIS) on the number of participants recruited into the trial (randomised vs not randomised), adjusted for the number of contacts made (continuous variable) and education status (categorised in a binary manner as university and pre-university qualifications). We replicated the same original frequentist analysis. Like Du et al.’s SWAT, results were similarly presented using ORs, *p*-values and 95% CIs to compare the recruitment intervention strategy.

### Bayesian SWAT analysis

For both the Du et al. and Mattock et al. SWAT, we performed Bayesian re-analysis for the same primary outcome of the original SWATs (recruitment into the main trial). For the Bayesian analysis of the Du et al. SWAT, an unadjusted Bayesian logistic model including intervention as the only covariate was fitted. For the Mattock et al. HSHS SWAT, a Bayesian logistic model was fitted, including intervention and adjustment for the same covariates used in the trial’s original frequentist analysis (number of contacts made and educational status).

In addition to selecting an analysis model suitable for the data at hand, a Bayesian analysis also requires prior assumptions about the intervention effect in the form of a prior distribution for the analysis model’s parameters. The prior is combined with the trial data in the analysis to form what is referred to as a posterior distribution for the intervention effect, and this results in an alternative interpretation for the intervention effect. It provides inference for the intervention effect in the form of probabilistic statements, e.g. the (posterior) probability that the intervention is more effective than the control (i.e. OR > 1). The prior may be uninformative if no previous beliefs or empirical evidence exists regarding the effect of an intervention. A non-informative prior typically results in low (or no) impact in parameters of the posterior distribution for a moderate sample size [7]. Or the prior may be formed based on the results of previous similar studies or other relevant information or elicited from clinical experts; this can result in a more efficient analysis by incorporating previous historical evidence in a single analysis.

For the primary Bayesian analyses of both SWATs, we initially adopted a non-informative uniform prior for the intervention effect (see Table 2). This prior is non-informative as it assigns equal probability to all possible values of the intervention effect within the specified range (Table 2). In subsequent sensitivity analyses, for each SWAT, we explored three further options for an informative prior to assess the impact of different priors and incorporation of previous evidence from previous SWATs on the intervention effect. For each SWAT analysed this included (i) a weakly informative prior [13], (ii) an informative prior based on the results of previously conducted similar SWATs [14] and (iii) a combined prior which was a weighted combination of the weakly informative and informative prior [15]. The informative prior for the Du et al. Bayesian SWAT analysis was informed by meta-analysis of two previous similar studies (Hutchinson 2007 [16] and Du 2008 [17]) which gave a pooled OR = 1.16, 95% CI 0.54–2.48 (see supplementary material for full meta-analysis results). The informative prior for the Mattock et al. HSHS Bayesian SWAT analysis was informed by meta-analysis of the same two previous similar studies and the Du 2009 SWAT (Hutchinson 2007 [16], Du 2008 [17] and Du 2009 [4]) which gave a pooled OR = 1.26, 95% CI 0.71–2.23 (see supplementary material for full meta-analysis results). These previous studies were identified from a Cochrane systematic review exploring video information interventions on trial recruitment [18]. Technical details on the construction of the priors are described further below in Table 2. Bayesian model diagnostics including convergence check (e.g. MCMC trace plots for the intervention effect) were assessed to confirm the adequacy of fitted models. Such diagnostics should always be performed when utilising Bayesian methods in practice.

**Table 2:** Prior distributions used in unadjusted and adjusted Bayesian logistic regression analysis

	**Unadjusted analysis**	**Adjusted analysis**
**Logistic model**	logit(p) = ꞵ_0_ + ꞵ_1_*x_1_	logit(p) = ꞵ_0_ + ꞵ_1_* x_1_ + ꞵ_2_* x_2_ + ꞵ_3_* x_3_
**Non-informative prior (uniform prior)**	ꞵ_i_ ~ Uniform (−15,15) for *i* = 0,1	ꞵ_i_ ~ Uniform (−15,15) for *i* = 0,1,2,3
**Weakly informative prior (Gelman prior **[13]**)**	ꞵ_0_ ~ student t (*µ* = 0, *σ = *10, df = 1) ꞵ_1_ ~ student t (*µ* = 0, *σ* = 2.5, df = 1)	ꞵ_0_ ~ student t (*µ* = 0, *σ* = 10, df = 1) ꞵ_i_ ~ student t (*µ* = 0, *σ* = 2.5, df = 1) for *i* = 1,2,3
**Informative prior (Sullivan prior **[14]**)**	ꞵ_0_ ~ Normal(µ = 0, σ^2^ = 1000) ꞵ_1_ ~ Normal(µ = (log(OR_upper) + log(OR_lower))/2, σ^2^ = (((log(OR_upper) - log(OR_lower))/(2 * 1.96))^2^), where (OR_upper, OR_lower) is the 95% CI for the odds ratio obtained from the meta-analysis^†^ of previous SWATs	ꞵ_0_, ꞵ_2_, ꞵ_3_ ~ Normal(µ = 0, σ^2^ = 1000) ꞵ_1_ ~ Normal(µ = (log(OR_upper) + log(OR_lower))/2, σ^2^ = (((log(OR_upper) - log(OR_lower))/(2 * 1.96))^2^), where (OR_upper, OR_lower) is the 95% CI for the odds ratio obtained from the meta-analysis^†^ of previous SWATs
**Combined prior (weighted weakly and informative prior) **[15]	ꞵ_i_ ~ 0.5 * ꞵ_i,Gelman prior_ + 0.5 * ꞵ_i,Sullivan prior_ for *i* = 0,1	ꞵ_i_ ~ 0.5 * ꞵ_i,Gelman prior_ + 0.5 * ꞵ_i,Sullivan prior_ for *i* = 0,1,2,3

Notation: x_1_ = intervention, x_2_ = number of contacts made, x_3_ = educational status, *p* = probability of enrolment, logit(p) = ln(p/(1-p)) = ln(Odds of enrolment), ꞵ_0_ = intercept i.e. log odds of enrolment for no intervention/no contacts/lowest educational status, ꞵ_1_ = intervention covariate = log odds of enrolment for intervention relative to control, ꞵ_2_ = no of contacts covariate = log odds of enrolment for one unit increase in no. of contacts made, ꞵ_3_ = educational status covariate = log odds of enrolment for higher educational statues relative to lowest, µ = location (centre), σ = scale, df = degrees of freedom. Note priors are applied after scaling all non binary variables (contact frequency, x_2_) to have mean 0 and standard deviation 0.5

^†^The full meta-analysis results of previous SWATs used to form the informative prior can be found in the supplementary material. For the Du et al. 2009 analysis OR_lower = 0.54 and OR_upper = 2.48; for the Mattock et al. 2020 HSHS analysis OR_lower = 0.71 and OR_upper = 2.23

An additional sensitivity analysis repeated the Bayesian analysis for the Mattock et al. HSHS SWAT using an unadjusted logistic model with priors constructed as per Table 2 column 2 (see appendix for results of this sensitivity analysis).

### ACCEPT analysis

For each Bayesian analysis ACceptability Curve Estimation using Probability Above Threshold (ACCEPT) was applied to aid interpretation [11]. This method was proposed by Clements et al. [11] to aid better integration and presentation of clinical trial findings for the treatment effect of interest. We explored novel application of how this method can also aid better interpretation of intervention effects in SWATs.

For each Bayesian analysis, ACCEPT uses the results (i.e. posterior distribution for the intervention effect of interest) to plot in a graph the probability of the intervention effect being above different threshold values on the y-axis for a range of different possible threshold values on the x-axis. The threshold values are referred to as “acceptability thresholds”. For our case studies, the intervention effect is expressed as an odds ratio for the outcome of interest for the intervention group relative to the control/standard group. This graphical representation readily enables reviewers to judge the strength of evidence for various intervention effect sizes, rather than focusing exclusively on binary trial conclusions. Thus, one can consider what would be acceptable thresholds to warrant the use of an intervention and weigh up the evidence in favour for this. ACCEPT curves are readily constructed after Bayesian analysis using the posterior distribution.

Clements et al. recommended that ACCEPT curve include a range of different threshold values for the intervention effect (acceptability thresholds) spanning unacceptable differences, zero (no effect), and a reasonable range of potential alternative acceptable values and acceptability thresholds for the 2.5th, 50th, and 97.5th percentile acceptability values. We therefore constructed ACCEPT curves for our SWAT case studies spanning intervention effects on an odds ratio scale from odds ratio = 0 to odds ratio = 1 to larger odds ratios beyond the 97.5th percentile acceptability value informed by the trial data. This resulted in going up to an odds ratio = 8 for the Du et al. 2009 SWAT analysis and odds ratio = 1.5 for the Mattock et al. HSHS SWAT analysis.

## Results

### Frequentist inference for Du et al. 2009 SWAT

Overall 10/98 (10%) participants given the video intervention were recruited, compared with 6/98 (6%) of participants given standard information. The OR for recruitment for the video intervention relative to standard information was 1.74, 95% CI: 0.60–5.03, *p* = 0.30, in favour of the video intervention but not significant. The large width of the 95% CI interval is consistent with a beneficial effect of the video intervention (upper bound 5.03), or a non-beneficial effect (lower bound 0.60).

### Bayesian inference for Du et al. 2009 SWAT

**Fig. 1:**
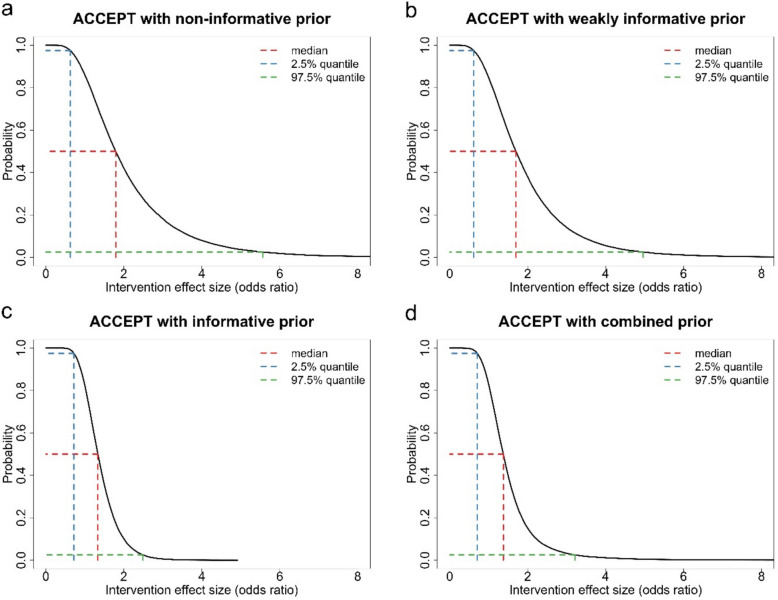
Bayesian ACCEPT analysis for Du et al. 2009 SWAT including primary and sensitivity analysis. OR represents odds of recruitment for video intervention relative to standard patient information

In the primary Bayesian re-analysis, utilising a non-informative uniform prior, the OR for recruitment for video intervention relative to standard information was 2.12, 95% CrI: 0.38–4.65. The posterior probability of the video intervention being effective (OR > 1) was 0.86 indicating a large probability of a beneficial effect. Figure 1a shows the ACCEPT curve for the primary Bayesian analysis. This illustrates the probability that the video intervention is better than the standard information for various different intervention effect sizes (as an OR) with the non-informative uniform prior. From this we can see a moderately high probability (>0.8) of the video intervention being effective in comparison to the standard information for various sized ORs > 1 up to OR’s around 1.5.

In sensitivity analysis using different priors, the result was robust; using the weakly informative prior gave a posterior probability of the video intervention being effective (OR > 1) of 0.85; using the informative prior, this was 0.82, and using the combined prior, this was 0.83 (see Table 3, Fig. 1b–d and supplementary material for more detailed sensitivity analysis results).

**Table 3:** Results of Frequentist and Bayesian SWAT analyses

Trial	Bayesian prior	Frequentist result			Bayesian result		
Analysis		OR	95% CI	*p*-value	OR	95% CrI	Probability OR > 1
** Du et al. 2009 SWAT **							
Primary analysis	Non-informative prior	1.74	0.60–5.03	0.30	2.12	0.38–4.65	0.86
Sensitivity analysis	Weakly informative prior				1.96	0.42–4.16	0.85
	Informative prior				1.40	0.63–2.31	0.82
	Combined prior				1.51	0.58–2.76	0.83
**Mattock et al. HSHS SWAT**							
Primary analysis	Non-informative prior	0.25^†^	0.10–0.62	0.003	0.26^†^	0.07–0.51	0.0005
Sensitivity analysis	Weakly informative prior				0.28^†^	0.07–0.54	0.0009
	Informative prior				0.78^†^	0.43–1.15	0.12
	Combined prior				0.31^†^	0.07–0.69	0.008

95% CI = 95% confidence interval, 95% CrI = 95% credible interval. OR = odds ratio for intervention compared to standard. ^†^adjusted OR for the number of contacts made (continuous variable) and education status (categorised in a binary manner as university and pre-university qualifications)

### Frequentist inference for Mattock et al. HSHS SWAT

Overall 10/50 (20%) participants given the video clip consented to take part in the trial, compared with 26/51 (51%) of participants given the traditional PIS. The adjusted OR for consent to take part in the trial for video clip relative to traditional PIS was 0.25, 95% CI: 0.10–0.62, *p* = 0.003, in favour of the traditional PIS. As reported in the original HSHS SWAT analysis report, “the odds of consenting to the trial were statistically lower in the video group compared to the standard PIS group” [5].

### Bayesian inference for Mattock et al. HSHS SWAT

**Fig. 2:**
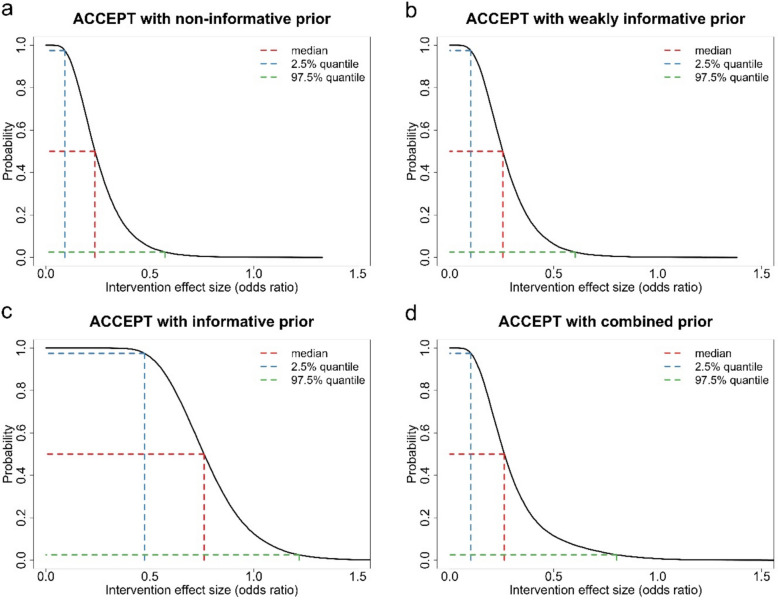
Bayesian analysis for HSHS SWAT including primary and sensitivity analysis. OR represents odds of recruitment for video intervention relative to standard patient information

In the primary Bayesian analysis, utilising a non-informative prior, the adjusted OR for consent to take part in the trial for video clip relative to traditional PIS was 0.26, 95% CrI: 0.07–0.51. The posterior probability of the treatment effect being effective (OR > 1) was 0.0005 indicating very little chance the video intervention was effective. Figure 2a shows the probability that the video intervention is better than the standard PIS for various different intervention effect sizes (as an OR) with a non-informative prior. From this we can immediately see the almost negligible probability that the video intervention is effective as there is an unapparent to the eye area under the curve for OR > 1. We in fact see there is very low probability that the intervention effect is OR > 0.8 (0.003).

In sensitivity analysis using informative priors, including the result of Du 2009 et al. in the prior, the probably of the video intervention being effective (OR > 1) varied slightly, but in all cases was still very small (see Fig. 2b–d). The largest probability for the intervention being effective was 0.12 when using the most informative prior. For the weakly informative prior this was 0.0009 and for the combined prior this was 0.008. In the additional sensitivity analysis excluding adjustment results were consistent (see Table 3 and supplementary material for additional sensitivity analysis results). A summary of all frequentist and Bayesian results can be found in Table 3.

## Discussion and conclusions

### Main findings

A Bayesian framework provided a different, accessible interpretation for the analysis of a SWAT. For the earlier Du et al. SWAT Bayesian analysis indicated a large probability of the video intervention being effective (OR > 1). However, for the later Mattock et al. HSHS SWAT, Bayesian analysis revealed a very small probability of the video intervention being effective in comparison to the standard information.

To reflect the uncertainty in results, Bayesian estimates are accompanied by 95% Credible Intervals (95% CrI). These represent the region that the intervention effect falls within with 95% (posterior) probability. Thus, for the latter, Mattock et al. HSHS SWAT it was identified that there was 95% probability that the video intervention effect (OR) lies within 0.07 to 0.51. In the frequentist analysis, uncertainty is represented by 95% confidence intervals (95% CI), which represent the region that, with a large number of repeated samples, 95% of such calculated confidence intervals would be expected to include the true value of the intervention effect, and this was 0.10–0.62. The probability statements about the intervention effect are directly provided by Bayesian analyses and are generally more intuitive to understand than CIs and *p*-values, which can be frequently misinterpreted [9].

When combining existing information for the intervention effect from previous similar SWATs into the Mattock et al. HSHS SWAT analysis, including those of Du et al., the evidence against the video intervention remained strong. In general, combining existing information where available can add efficiency into the research process and is particularly advantageous for SWATs where the sample size is limited. A Bayesian framework offers the ability to do this through incorporating previous SWAT results within the analyses prior. We used previous similar SWATs identified from a previous Cochrane systematic review exploring video information interventions on trial recruitment [18]. No formal criteria currently exist however to guide which previous SWATs are adequately similar to use to form informative analysis priors. This is indeed an area worthy of future research. Literature review and previous systematic reviews can help guide researchers, but careful consideration as to which SWATs are adequately similar is required by researchers in each individual context. As explored here, meta-analysis of studies judged adequately similar can be useful to form informative priors. We note that where there are concerns about the presence of heterogeneity between previous and current SWATs, the information from previous similar SWATs can be down weighted in the prior [19]. The use of a combined prior can be adopted to do so. We applied combined priors with equal weight given to the informative and weakly informative prior, however alternative weights can be applied as appropriate in different settings and may provide useful sensitivity analysis.

The results shown here illustrate how the prior does impact inference. We recommend sensitivity analysis around the prior be conducted when Bayesian methods are adopted to assess the consistency of results under various assumptions. In this case study, although results were robust to the prior (whether it was informative or uninformative), in other settings the prior may have a bigger impact on conclusions.

ACCEPT plots provided further insight on the probability of the intervention effect taking different values. This approach entails plotting the probability of the intervention effect being above different numerical values for a range of different possible values. This adds value and enables trialists to move away from making rigid binary conclusions based on statistical significance.

In SWATs, this may be partially useful where the SWAT has not been powered to detect a specific effect size and is reliant on the main host trials sample size. Additionally, for a SWAT, it may be hard to establish what a minimally important difference to detect is. Thus, one can consider what would be acceptable thresholds to warrant the use of an intervention and weigh up the evidence in favour for this. What is considered acceptable may vary for different stakeholders, thus different stakeholders can examine the ACCEPT plot and what is important for them. Different effect sizes can be examined and probabilities of obtaining that effect presented for these for a single SWAT. Trialists using the results of a SWAT to plan methods for future trials can then weigh up the costs associated with a particular trial strategy to determine whether the probability of effectiveness is adequately large to warrant utilising the trial strategy in a particular trial setting.

### Strengths and limitations

A limitation of this study is that we focussed on only two case studies exploring the effect of a video intervention on trial recruitment. This approach was chosen to clearly illustrate Bayesian inference and ACCEPT plots in a single intervention setting to demonstrate proof of concept and how Bayesian methods can incorporate previous evidence, culminating in an effect estimate based on the totality of evidence collected at a specific date. However, the general analytical principles are widely applicable. It is important to further explore the use of Bayesian inference in different contexts for more SWATs. In this setting, the conclusion from both the frequentist and Bayesian inference of the Mattock et al. SWAT incorporating data from the previous SWATS including Du et al. was clear-cut on no intervention effect. It is conceivable that the Bayesian approach with ACCEPT plots will be more advantageous where the frequentist conclusion is more marginal (e.g. *p*-value close to 0.05). This can be seen through comparing Figs. 1 and 2, where arguably Fig. 1 is more informative. ACCEPT plots enable more nuanced interpretation of the probability of the intervention effect being greater than more than one specific value. The methods are not limited to a binary outcome and can be used with all other outcome types. They can be implemented with an analysis model suitable for the type of outcome variable at hand, e.g. linear for continuous, Poisson for count etc.

Our focus was on contrasting the results of frequentist and Bayesian analyses for two SWATs, based on the information currently available at the time of the original SWATs. A Cochrane systematic review was used to inform this [18]. Thus, the prior information included in the Bayesian analysis does not include information published after the HSHS SWAT publication and is not necessarily comprehensive finding for the effectiveness of a video intervention on trial recruitment. Moreover, we did not account for potential differences between previous and HSHS SWATs when we constructed the informative priors in our sensitivity analysis, but as expected, the impact of a prior constructed with down weighted information is between those of non-informative and informative priors. Existing methods for capturing the heterogeneity between previous and current studies can be found in Schmidli et al. and Ibrahim et al. [19].

### How this study compares with others/research in context

We have not found any SWATs utilising Bayesian methods, with or without ACCEPT plots, for their analysis. A search of the online database PubMed on 12th February 2026 including “Bayesian” and “Study within a trial” or “SWAT” produced no relevant results. This may be due to a lack of awareness or knowledge on the part of those doing analysis of SWATs. ACCEPT has been previously advocated for use in clinical trials reporting [11] and we hope this case study provides an example of the advantages for using such an analytical framework for SWATs. It is particularly useful where historical data is available to combat the issue of the lack of statistical power and reduce uncertainty in estimation.

To account for the lack of statistical power, as discussed by Arundel et al. [20], SWATs are often combined in a meta-analysis once data is available from sufficient similar SWATs. However, this can take many years to reach a consensus on effectiveness, especially if a single binary criterion for efficacy is explored in a frequentist analysis. By using a Bayesian framework, one is able to easily incorporate previous evidence and estimate the most up-to-date probability that an intervention is effective. ACCEPT plots provide further nuances in the interpretation based on exploring the probability of the intervention being effective for various different effect sizes.

If Bayesian methods are to be used for the analysis of SWATs during the planning stages of a SWAT, Bayesian methods can also be used to design the SWAT. Although not explored here, Bayesian methods can be used to estimate the sample size required for a SWAT. Or in a particular host trial setting, where the SWAT is naturally restricted by the size of the host trial, Bayesian methods can be used to establish whether it is worth undertaking a SWAT given the available sample size. This may be particularly of value when a SWAT is being introduced part way through the host trial such that its sample size is further restricted below the host trial’s sample size. This was the case for the HSHS SWAT which was introduced part way through the conduct of the HSHS main trial. The use of simulations and Bayesian methods can reveal the potential posterior probability of finding a successful intervention in a SWAT at the design stage for a given sample size. Further exploration on the use of Bayesian methods to design SWATs is warranted in future work.

## Conclusions

Bayesian inference and ACCEPT analyses offer solutions to challenges experienced in the analysis and interpretation of SWATs. We advocate the use of these analytical methods in SWATs and provide code to readily implement these methods. Greater use of these analytical approaches within SWATs will lead to a more accessible, improved evidence base on how to effectively conduct RCTs.

## Supplementary Information

Additional file 1. Supplementary material.

Additional file 2.

## Data Availability

Data is available upon reasonable request.
