# Supplementary material for: The use of Bayesian methods for the analysis of Studies Within A Trial: a proof-of-concept case study
Source: Trials. 2026 Apr 24;27:420. doi: 10.1186/s13063-026-09726-z (PMC13244628; doi:10.1186/s13063-026-09726-z)
Supplement: Supplementary file 1 — Additional file 1. Supplementary material. [file 13063_2026_9726_MOESM1_ESM.docx]

**Supplementary file 1 – additional results**

**Meta-analysis of previous similar SWATs for Du et al 2009 analysis**


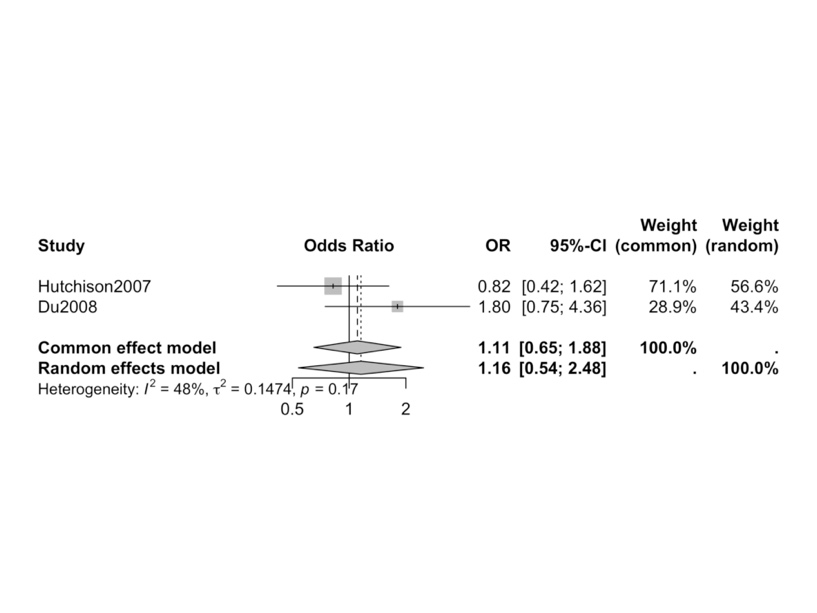


Figure S1 - Meta-analysis of previous similar SWATs to form priors for Du et al 2009 analysis

**Meta-analysis of previous similar SWATs for Mattock et al 2020 HSHS analysis:**


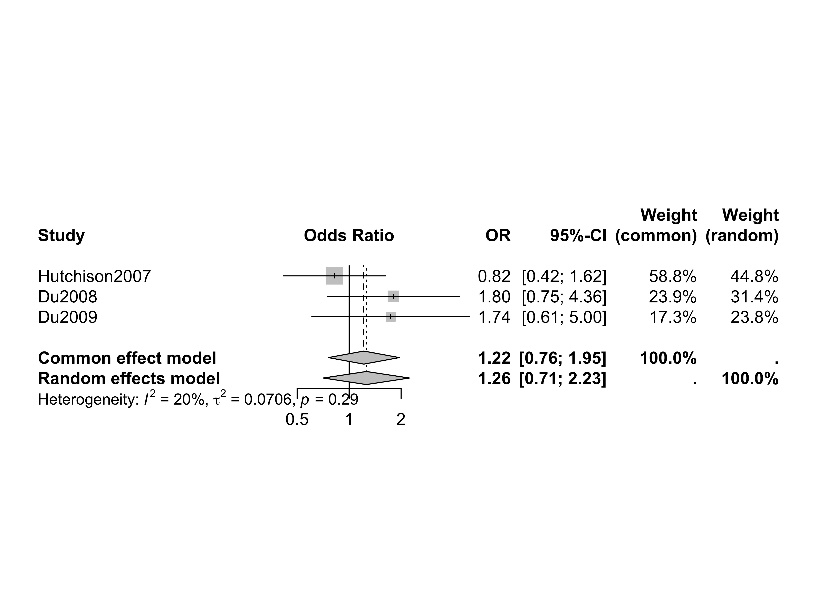


Figure S2 - Meta-analysis of previous similar SWATs to form priors for Mattock et al 2020 analysis

**Additional results for Du et al SWAT sensitivity analysis**

Weakly informative Gelman prior:

Pr(OR > 1) = 0.85

MMSE estimate = 1.96

95% CI = (0.42, 4.16)

Informative Sullivan prior:

Pr(OR > 1) = 0.82

MMSE estimate = 1.40

95% CI = (0.63, 2.31)

Combined prior:

Pr(OR > 1) = 0.83

MMSE estimate = 1.51

95% CI = (0.58, 2.76)

**Additional results for HSHS SWAT sensitivity analysis – adjusted**

Weakly informative Gelman prior, adjusted analysis:

Pr(OR > 1) = 0.0009

MMSE estimate = 0.28

95% CI = (0.07, 0.54)

Informative Sullivan prior, adjusted analysis:

Pr(OR > 1) = 0.12

MMSE estimate = 0.78

95% CI = (0.43, 1.15)

Combined prior, adjusted analysis:

Pr(OR > 1) = 0.008

MMSE estimate = 0.31

95% CI = (0.07, 0.69)

**Additional sensitivity analysis for HSHS SWAT – unadjusted**


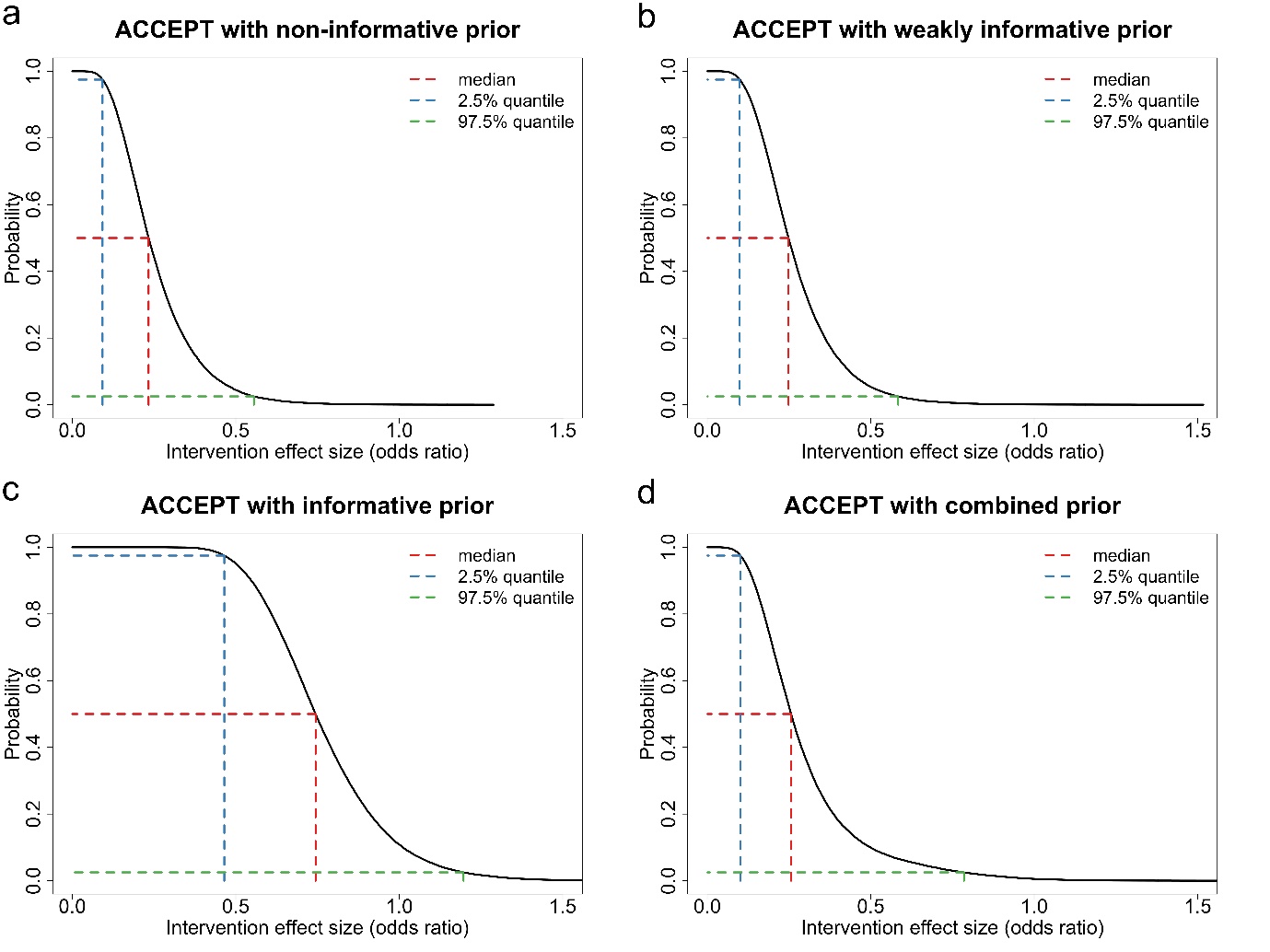


Figure S3 - Bayesian unadjusted analysis for HSHS SWAT including primary and sensitivity analysis. OR represents odds of recruitment for video intervention relative to standard patient information.

Non-informative Uniform prior:

Pr(OR > 1) = 0.0004

MMSE OR estimate = 0.26

95% CrI = (0.07, 0.50)

Weakly informative Gelman prior:

Pr(OR > 1) = 0.0006

MMSE estimate = 0.27

95% CrI = (0.08, 0.53)

Informative Sullivan prior:

Pr(OR > 1) = 0.11

MMSE estimate = 0.76

95% CrI = (0.43, 1.14)

Combined prior:

Pr(OR > 1) = 0.006

MMSE estimate = 0.30

95% CrI = (0.07, 0.64)
